# Adults Aged 75+ Happy in Conventional Dwelling or Independent Living Facility but Associated With Thriving and Ageism

**DOI:** 10.1177/01640275251328591

**Published:** 2025-03-24

**Authors:** Mélanie Levasseur, Daniel Naud, Martine Lagacé, Émilie Raymond, Mélissa Généreux, Sébastien Lord, Marie-Ève Bédard

**Affiliations:** 1 Faculté de médecine et des sciences de la santé, 7321 Université de Sherbrooke, Sherbrooke, QC, Canada; 2 Centre de recherche sur le vieillissement, 142379 CIUSSS de l’Estrie-CHUS, Sherbrooke, QC, Canada; 3 Faculté des arts, 6363 Université d’Ottawa, Ottawa, ON, Canada; 4 Faculté des sciences sociales, 4440 Université Laval, Quebec, QC, Canada; 5 Facultad de Derecho y Humanidades, Universidad Central de Chile, Santiago, Chile; 6 Faculté de l’aménagement, 5622 Université de Montréal, Montreal, QC, Canada; 7 Centre collégial d’expertise en gérontologie, 155709 Cégep de Drummondville, Drummondville, QC, Canada

**Keywords:** well-being, retirement homes, quality of life, sheltered housing, aging in place, fulfillment

## Abstract

This study aimed to compare levels of happiness of older women and men living in conventional dwellings (CD) and independent living facilities (ILF), and examine happiness’ associations with thriving, social participation, community integration and ageism (self-directed and discrimination). A cross-sectional survey was conducted with a random sample of 509 older adults in CD and 470 in ILF in Quebec, Canada. Participants’ mean age was 82.22 ± 5.35, and two-thirds were women. Levels of happiness were similar in both sexes and settings. Greater happiness was associated with greater thriving for all (β = 0.28–1.48), social participation for women in CD (β = 0.67), community integration in CD (β = 0.42 for women and 1.18 for men), and reduced ageism, i.e., discrimination for women in CD (β = −1.02) and men in ILF (β = −0.28), and self-directed for men in CD (β = −0.21). The findings demonstrate that happiness was associated with factors related to the living environment and could be enhanced through targeted interventions.

## Introduction

In recent decades, major advances in the fields of medicine and public health, and the adoption of healthier lifestyles have contributed to significant gains in life expectancy and healthy aging (for Economic Co-operation and Development [OECD], 2017). In Quebec (Canada), which is located in eastern North America, women and men could expect to live in good health until the age of 72.0 and 71.2, respectively, ranking them first and second compared to older adults in other Canadian provinces in 2015–2017 (Statistics Canada, 2017). Despite this progress, the health characteristics of Canada’s aging population are increasingly heterogeneous (Nguyen et al., 2021). For example, one third of Quebecers aged 75 and over (32.8%) had at least one disability.

According to the World Health Organization’s (WHO) definition of health as “*a state of complete physical, mental and social well-being and not merely the absence of disease or infirmity*” (WHO, 1946, p. 1), maintaining and improving well-being is an essential goal (WHO, 2020). The objective dimension of well-being refers to the conditions that enable people to carry out their activities, and its subjective dimension refers to people’s cognitive and emotional reactions, i.e., satisfaction with life, positive and negative affects, and quality of life based on capabilities (Diener et al., 1999; OECD, 2013). While *life satisfaction* is a person’s assessment of their quality of life and achievements, affects are a person’s emotional states at a given moment in time (OECD, 2013). These affects can be positive (happiness, enjoyment of life, contentment, etc.) or negative (sadness, anger, anxiety, etc.). *Quality of life based on capabilities* represents the functioning and realization of a person’s potential according to autonomy, competence, goals, purposefulness, and altruism, in relation to the person’s capabilities (Huppert & So, 2009; OECD, 2013). Results from the French Eurobarometer survey conducted between 1975 and 2000 (Afsa & Marcus, 2008) showed a decrease in happiness levels in the 20-to-50 year-old age range, followed by a gradual rise, reaching a peak between 65 and 70 years old, with a sharp decline in later years; these results still stand, even after adjusting for age, generation, and survey timing. According to longitudinal studies conducted in the United States since 1970 (Stevenson & Wolfers, 2009), it was more common for women to report being very happy than men. At the end of 1990, this trend shifted, and the happiness of women decreased, predominantly when aged 60 and older. Based on results from the 2016 Canadian General Social Survey, life satisfaction increased with age and older women were more likely than men to report better life satisfaction (Uppal & Barayandema, 2018).

The happiness of older adults is influenced by a person’s living environment. Ecological models of human development, notably the Human Development Model-Disability Creation Process (HDM-DCP; Fougeyrollas et al., 2019) and Bronfenbrenner’s Ecological Systems Theory (EST; Bronfenbrenner, 1979; Merçon-Vargas et al., 2020), offer comprehensive frameworks to understand the potential mechanisms underlying the happiness of older adults within the context of their living environment. The EST’s *microsystem* is the most immediate social environment, where individuals can experience supportive and meaningful activities and interactions with family, friends, and caregivers. Within a living environment that fosters these interactions, a person can develop a sense of purpose and well-being. The *mesosystem* involves the interactions between the person’s multiple social environments such as family, healthcare providers, neighbors or community organizations, which interact to create a supportive environment. The HDM-DCP adds that environmental factors and life habits can support a person with declining capacities and create opportunities for meaningful social participation. In the mesosystem, family members, neighbors or caregivers can hold ageist attitudes, even unconsciously, which may act as a barrier to engage in social activities, and lead to isolation and decreased happiness. Additionally, the HDM-DCP’s environmental factors emphasize that a community that proposes inclusive programs and accessible activities can foster participation, contributing to social integration and countering ageist attitudes (Poulin et al., 2024). From this perspective, the happiness of older adults is intertwined with factors from the living environment, adapting to the person’s capacities and facilitating their social inclusion and well-being.

In Quebec, nearly one in five people aged 75 and over (17.0%) lived in an independent living facility (ILF), where the average age was 83 (Canada Mortgage and Housing Corporation [CMHC], 2021), and over two thirds (71.0%) of all residents were women (Statistics Canada, 2021), which is a higher proportion than in the total population aged 75+ (56.5%; Institut de la statistique du Québec, 2022). ILF are homes for autonomous or semi-autonomous older adults, generally managed by a private company, which also provides services (meals, assistance, care, security and/or recreation; Plourde & Pratte, 2021). When major disabilities or life events occur, people must make important decisions about their living environment, such as adapting their home, getting home care support or moving to an ILF. The transition to an ILF, when this is the choice of the older adult, is associated with good quality of life and a sense of being at home (Street et al., 2007). According to a 2021 survey of 1028 respondents, the majority of Quebecers aged 70+ (53%) reported a more negative perception of ILF compared to before the COVID-19 pandemic, and nearly a fifth (19%) had postponed their plans to move to an ILF (Bail, 2021). Many community-dwelling older adults are hesitant to change their living environment (Lord & Després, 2011; Ossokina & Arentze, 2020), and may prioritize the comfort of staying in a familiar and supportive environment when their income is sufficient and their physical health permits (CMHC, 2012).

### Residential Setting

To our knowledge, few studies have compared happiness or its components (life satisfaction, affects and quality of life) in CD and ILF. An American study of older adults living in CD (*n* = 119; 83.4 ± 6.5 years) and ILF (*n* = 104; 83.6 ± 6.6 years) showed that participants’ life satisfaction (*p* = .78) and happiness (*p* = .14) were similar across these living environments (Jeste et al., 2019). Despot Lučanin et al. (2020) also observed similar life satisfaction (*p* > .05) among Croatian older adults, whether they lived in ILF (*n* = 101) or CD (*n* = 101), and regardless of sex. Conversely, Lynch (2012) found greater life satisfaction among older adults in Chicago living in ILF (*p* = .01; *n* = 146) compared to those living in CD (*n* = 200), but no difference between women and men; however, both studies used convenience sampling. In Quebec, nearly half (46%) of the 1200 respondents to a 2017 survey reported being happier since living in an ILF, compared to their previous CD (Léger Marketing, 2017), despite happiness being measured by a single question and with no knowledge of when they moved. Finally, according to this same survey, loneliness, difficulty maintaining one’s home, and functional dependence were the main reasons given for moving into an ILF, underlining older adults’ need for support and interaction.

### Thriving

Within the living environment, a number of personal and environmental characteristics are associated with happiness (Diener et al., 1999). The HDM-DCP and the EST highlighted the immediate personal experiences within a person’s social environment, notably the person’s thriving (Bergland & Kirkevold, 2001) and social participation (Dupuis-Blanchard et al., 2009). By contrast, ageism (Menkin, 2017) and the sense of belonging to a community (Menkin, 2017) are environmental factors operating at the mesosystem level and shape the experience of thriving and social participation, as barrier and facilitator, respectively. Thriving is a dynamic and voluntary process of interactions between a person and their context over time, which facilitates the simultaneous improvement of the person and their environment (Bundick et al., 2010). Thriving requires the individual to determine and achieve their intrinsic aspirations (Diener et al., 1999) by mobilizing or adapting the social and material resources in the environment (Bergland & Kirkevold, 2001). According to a Swedish study, the thriving of older adults living in a CD (*n* = 1850; 82.9 ± 6.4 years) was similar to that of those living in an ILF (*n* = 1955; 83.1 ± 6.5 years; *p* = .28), and no difference was found between women and men (Corneliusson et al., 2020). As older women were more functionally dependent than men, they might have needed additional assistance with daily activities, and their thriving could have had a greater impact on their happiness. Better adaptation of the place of residence to their abilities enabled ILF residents to increase their social participation and to reduce the risk of fatigue, weakness or depressive symptoms (Haight et al., 2002).

### Social Participation

Defined as “a person’s involvement in activities providing interactions with others” (Levasseur et al., 2010, p. 2146), “in community life and in important shared spaces, evolving according to available time and resources, and based on the societal context and what individuals want and is meaningful to them” (Levasseur et al., 2022, p. 8), social participation is associated with greater satisfaction with life (Cantor & Sanderson, 1999). A study of 2475 Swiss aged 65+ showed that a higher level of participation was associated with greater life satisfaction (*p* < .01), controlling for health and socioeconomic characteristics (Baeriswyl & Oris, 2021). According to a recent study, older Japanese (*n* = 143) living in an ILF were more likely to engage in hobbies, as well as physical and learning activities, than those living in a CD (*n* = 398; Kawaguchi et al., 2024) because the staff fostered social participation. According to another cross-sectional Swedish study, social participation (*p* < .001) and health (*p* < .001) were lower among ILF respondents than those in CD (Corneliusson et al., 2019), reflecting ILF residents’ greater need for support. According to Canadian women aged between 79 and 90 (mean = 84.2), the transition to an ILF helped increase their social participation, especially their physical activity with others, and their functional independence, despite reduced control over certain aspects of their lives (such as nutrition; Haney et al., 2018).

### Ageism

Ageism is the combination of stereotyping (how we think), prejudice (how we feel) and discrimination (how we act) based on age (WHO, 2021). An environment is inclusive if it promotes equality, full participation, well-being and integration into the community for older people. Ageism can interact with personal development associated with thriving, and can contribute to social isolation and loneliness, through age discrimination and self-directed stereotyping (Shiovitz-Ezra et al., 2018). The experience of age discrimination is associated with lower life satisfaction (*p* < .01) among aging Canadians (*n* = 15,759), particularly women aged 55–64 (Browning et al., 2020), as well as less happiness among aging Australians (*n* = 2119), especially younger-old and men (Lyons et al., 2018), implying that the intersection of gender and age influences the association between discrimination and life satisfaction. According to a longitudinal study of 3034 aging Americans, prolonged experience of age discrimination predicted a decline in mental health, reduction in positive affects, and further limitations in instrumental activities of daily living (IADL) (Stokes & Moorman, 2020). According to American ILF residents (*n* = 14), their relocation contributed to a sense of feeling useless and incompetent, potentially developing self-directed stereotypes (Jungers, 2010). Moreover, as ILF residents in Los Angeles (*n* = 81) reporting positive self-directed stereotypes on aging engaged in more social interactions than their negative counterparts (Menkin, 2017), ageism might moderate the associations between happiness and social interactions. However, residents may also express stereotypes or ageist attitudes towards other residents, particularly those with disabilities, contributing to reduced quality of life (Bodner et al., 2011).

### Community Integration

*Community integration* is a process comprised of four main dimensions: support, autonomy, occupation and assimilation (McColl et al., 2001). Support represents exchanges and relationships with family members, friends and neighbors. Autonomy in relation to living conditions means that people can decide for themselves, according to their interests, and participate socially without help. Occupation (education, work, volunteering) generates the feeling of contributing to one’s community, and of being rewarded and respected for it, and leisure enables one to take advantage of the opportunities for participation in one’s community. Finally, assimilation is based on knowledge of and compliance with community rules and norms, a sense of acceptance and the ability to orient oneself (McColl et al., 1998). Community integration contributes to the creation of a supportive environment, socially and physically, and provides more opportunities for meaningful activities with others (Scharlach & Lehning, 2013), consistent with the HDM-DCP and the EST. According to data collected from 4541 Quebecers aged 60–106 and living in CD, a greater sense of belonging (i.e., subjective experience of integration; McColl et al., 2001) is associated with more social participation (*p* < .001), especially when participants reported greater resilience (Levasseur et al., 2017). Based on this association with social participation, community integration could act as a moderating factor between social participation and happiness.

As suggested by the HDM-DCP (Fougeyrollas et al., 2019), well-being can have a strong gender and sex component, as sex characteristics and gender identity intersect with social attitudes and expectations, as supported by differences in health trajectories (Crimmins et al., 2011), social support (Levasseur et al., 2011), barriers for social participation (Naud et al., 2020), and life satisfaction (Pinquart & Sörensen, 2000). Based on a study of the Gallup World Poll’s global data (Joshanloo & Jovanović, 2020), inferior life satisfaction was observed in older women (65+), and, according to a meta-analysis, older women report inferior happiness (56 studies) and life satisfaction (176 studies; Pinquart & Sörensen, 2001). In addition, the meta-analysis found stronger association between life satisfaction and social support in older women, and income and education in older men. Other studies showed that women (Maurya et al., 2022) or men (Rippon et al., 2014) are as likely to experience age discrimination, and that older men were more prone to avoid contact with peers from the same age-group and have self-directed ageist attitudes (Bodner et al., 2012). Numerous gender-based associations highlight the need to better understand the potential distinct pathways that may explain the happiness of older women and men.

In sum, fostering happy and active aging underlines the importance of diverse living environments and places of residence, adapted to facilitate older adults’ thriving and social participation, free of ageism and supporting community integration. Happiness is an important and desirable component of people’s lives, as well as being associated with physical and mental health. However, little is known about the factors in the living environment that influence a person’s happiness, or how gender associates with these factors. To guide older women and men in making decisions regarding their choice of living environment, it is essential to have a better understanding of the associations between happiness, thriving, social participation, ageism, and a sense of belonging to the community of older women and men living in CD or ILF. The purpose of the present study was thus to: (1) compare happiness, thriving, social participation, ageism, and community integration between older adults living in CD and ILF, considering sex, and (2) examine the associations between, on the one hand, happiness and, on the other, thriving and social participation while testing the moderating effect of ageism and community integration.

## Research Design

### Design

**Figure 1.**
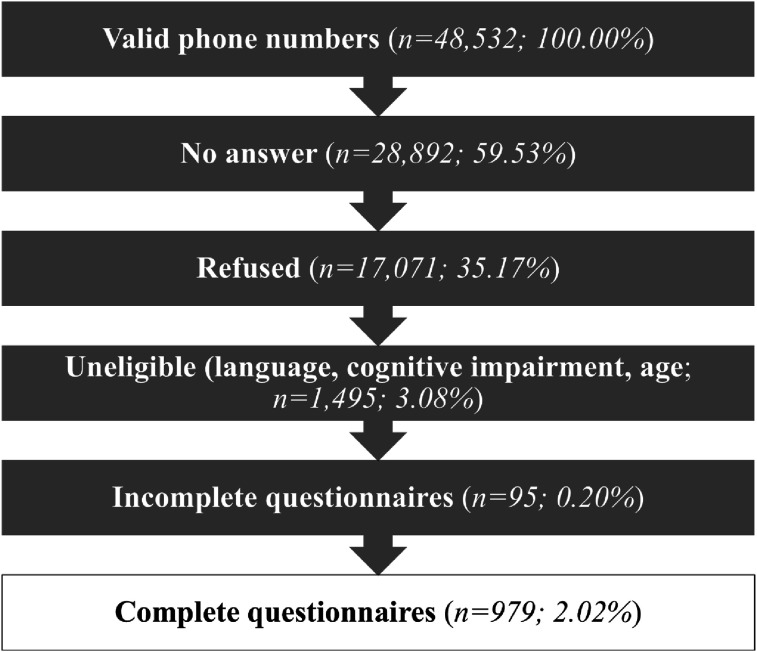
Summary of exclusion and refusal rates.

To address the purpose of this study, data from a cross-sectional survey were used. First, this survey was submitted to older adults living in conventional dwellings (i.e., apartment, condominium, cooperative, detached single-family home, semi-detached, duplex, row house or intergenerational) and ILF between July and September 2023. A CD is a place of residence that does not provide access to aid, assistance or health care services (with the exception of home help); it excludes low-income housing with services, non-profit organizations with services, ILF, and institutional and non-institutional housing resources (Conseil des aînés, 2007). An ILF is a residential building occupied by people aged 65 and over, where services are provided (Éditeur officiel du Québec, 2023). Only autonomous ILF (and floors reserved for autonomous residents in mixed ILF) were eligible for inclusion in this study. To be eligible, respondents had to: (1) be aged 75 or over; (2) be functionally autonomous (self-assessed with the question: “Are you able to carry out your daily activities without the help of another person?”); (3) not be cognitively impaired (seemed to understand the questions in the survey); (4) communicate orally; and (5) be able to speak French. A sample of 979 Quebec respondents (509 in CD and 470 in ILF) was collected (Figure 1); this sample size provided 80% power to detect, on the happiness measure, a standardized difference of 0.179. According to Cohen (2013), this difference between two means was considered small, using a two-tailed independent-samples *t* test and an alpha level of 0.05. Additionally, when stratified by sex and residential setting, the sample size provided 80% power to detect standardized differences of 0.262 and 0.292 between women and men living in CD and ILF, respectively. Random recruitment in ILF was carried out from two sources: (1) a denominalized list of 8,472 landline and cellular telephone numbers (out of a population of 13,200 residents) provided by our partners; (2) a denominalized list of 304 ILF addresses registered on the *Regroupement québécois des résidences pour aînés* (out of 800); then the numbers corresponding to these addresses were obtained from online registers (e.g., Canada411). Random recruitment of older adults living in CD was carried out by a polling firm, based on the random dialing of landline telephone numbers. The random dialing may also have allowed the recruitment of respondents living in ILF. This study was approved by the Research Ethics Committee of the Centre intégré universitaire de santé et de services sociaux (CIUSSS) de l’Estrie - Centre hospitalier universitaire de Sherbrooke (CHUS; #2022–4585).

### Variables

Happiness was estimated using three instruments and considering the cognitive and affective dimensions. The cognitive dimension of happiness was estimated using the French version of the *Satisfaction With Life Scale* (SWLS; Blais et al., 1989; Diener et al., 1985). The SWLS measures the convergence or divergence between a person’s aspirations and achievements using five questions (4-point Likert scale), with a final score ranging from 5 (dissatisfaction) to 20 (satisfaction). Validated with older Quebecers (*n* = 121; mean age = 76.3) living in an ILF, conventional home or apartment, the SWLS has good internal consistency (Blais et al., 1989). The instrument also exhibits good construct validity, being correlated with self-esteem (r = 0.22; *p* = .02), social anxiety (r = −0.19; *p* < − 0.03; Blais et al., 1989), joy (r = 0.61; *p* < .01), sadness (r = −0.55; *p* < .01) and anger (r = −0.36; *p* < .01; Ricard-St-Aubin et al., 2010). The affective dimension was estimated with questions selected by the OECD (2013), including four questions measuring positive affect (pleasure, calm, joy and laughter) and six measuring negative affect (worry, sadness, depression, anger, stress and fatigue). Respondents were asked to report the extent to which they had felt each affect during the previous day (0 [not at all] to 10 [all day]). The average of the scores determined the two affective states, and the values of the negative affect were inverted to correlate with happiness. Positive affect was positively correlated with the SWLS (r = 0.42; *p* < .05) and negative affect was negatively correlated (r between −0.25 and −0.32; *p* < .05), showing good construct validity (Cheung & Lucas, 2014). Also for the affective dimension, a final instrument considered quality of life in relation to abilities, i.e., the ICEpop CAPability measure for Older people (ICECAP-O; Adams et al., 2019). The ICECAP-O measures the person’s psychological functioning and intrinsic aspirations, using five questions (relating to love and friendship, the future, self-worth, pleasure, and independence; 4-point Likert scale; Proud et al., 2019). It has good construct validity, being correlated with a quality of life measure (EQ-5D-5L; r = 0.68; *p* < .001) and with the SWLS (*r* = 0.82; *p* < .001; Hackert et al., 2017). The sum of the five aspects determines the overall quality of life (range 5 [poor] to 20 [excellent]). Positive correlations between happiness dimensions allow scores to be aggregated into a composite happiness value for analysis. Final scores for each instrument (SWLS, Positive and Negative Affects, and ICECAP-O) were standardized from 0 to 1, averaged, and reported on a 0–100 scale, for ease of interpretation.

Thriving was assessed with the TOPAS-F, the French version of the short version of the “Thriving of Older People Assessment Scale” (TOPAS; Corneliusson et al., 2020), an instrument that specifically measures the thriving of older adults in relation to their living environment (e.g., “I try to see the positive sides of being in this dwelling” and “I receive help when I wish”). The TOPAS was translated from English to French, followed by a back-translation from French to English, to compare the two versions. The TOPAS-F includes 15 questions (6-point Likert scale) and shows good face validity with the theory of thriving, and good internal consistency (*α* = 0.90; Baxter et al., 2019). The final score is the sum of the 15 responses (15 [not thriving] to 60 [thriving]; Baxter et al., 2019).

Social participation was measured by self-reported frequency of involvement in 10 activities with others outside the home: visiting family or friends; pursuing a hobby; participating in activities at a community or recreation center; shopping; going to a restaurant, pub or café; participating in a self-help or discussion group; attending a library or cultural center; attending sporting or cultural events; taking classes; and volunteering, including with religious groups. Internal consistency was satisfactory (α = 0.85; (Levasseur et al., 2011, 2015; Richard et al., 2013). Responses were converted into monthly frequency of participation (“at least once a day” = 20; “at least once a week” = 6; “at least once a month” = 2; “at least once a year” = 1; and “never” = 0; Levasseur et al., 2011, 2015; Naud et al., 2019). The sum of the activities gives a total social participation score representing the number of community activities per month.

Ageism was estimated using the Everyday Ageism Scale (EAS), a 10-item multidimensional tool measuring discrimination (experience of ageism or ageist messages; 7 items) and stereotyping (internalized ageism; 3 items) on a 4-point Likert scale (Allen et al., 2022). Responses were summed to obtain scores for age discrimination (7–28) and self-directed ageist stereotypes (3–12). The EAS showed satisfactory internal consistency (α = 0.77).

Community integration was estimated using the French version of the Community Integration Measure (CIM; Lefebvre & Vanier, 2004; McColl et al., 2001), a 9-item questionnaire (4-point Likert scale), measuring two dimensions: community belonging (e.g., *“I feel accepted in this community”*) and community participation (e.g., *“I know the rules of this community and can adapt to them”*). The CIM has good internal consistency (α = 0.87) and good content (Lefebvre & Vanier, 2004) and convergent validity (McColl et al., 2001). Two items on feelings of safety were added: “I feel very safe walking alone in my community” and “When I’m outside at night when it’s dark, I feel very safe”. The sum of the scores determines the final score (11 [no integration] to 44 [excellent integration]).

Several personal characteristics were used as control variables. The continuous control variables were age, and number of people living with the respondent. In addition, four questions assessed the neighborhood’s friendliness in terms of: (1) obtaining quality, affordable food, (2) finding a variety of shops and services, (3) having access to interesting leisure activities, and (4) having access to physical activities or sports (4-point Likert scale). Responses were summed to estimate access to services and activities (4 [low access] to 16 [optimal access]), with satisfactory internal consistency (α = 0.74). Categorical control variables were self-identified gender (including non-binarity), self-reported physical and mental health status, highest level of completed education, marital status, employment status and income situation. An estimate of the number of ILF residents was asked for, as well as dwelling type (apartment, condominium, co-op or house) and home occupancy status (owner or renter), for respondents living in an ILF or CD, respectively. No identifiable data, for example the ILF’s name, were collected.

### Analysis

Respondents were described using frequencies and percentages for categorical variables, and means and standard deviations for continuous variables. Means adjusted for age, living with another person, and physical and mental health, were reported for happiness and its four subscales, as well as for associated factors. Respondents were compared by living environment and gender, using chi^2^ and independent-samples t-tests with Bonferroni correction. Linear regression analyses, stratified by gender and place of residence, examined associations between happiness and the independent variables, fulfillment and social participation, testing for the moderating effects of ageism and community integration, and controlling for respondents’ characteristics. Variables were selected by elimination, retaining only those with a significant association (*p* < .05). A non-significant variable was retained in the model if it interacted significantly (*p* < .05) with another variable. Four models are reported: (1) main effects for thriving and social participation; (2) main effects for thriving, social participation, and both main and interaction effects for ageism (self-directed and discrimination) and community integration; (3) inclusion of personal characteristics as control variables (age, living with someone, physical and mental health, income situation, and services); and (4) final model with main and interaction effects, and control variables. The standardized coefficients are reported.

Mean-value imputation was applied to all measures when a respondent had more than 75% valid responses on an instrument. Prior imputation, the missing completely at random assumption was tested for all measures to be imputed using the Little’s chi-square test (Li, 2013; Little, 1988). Moreover, sensitivity analyses replicated the models using non-imputed measures (*n* = 321 in CD and *n* = 360 in ILF), showing small to no differences in terms of size effect and significance. To normalize the distribution, social participation was transformed by its square root (Tabachnick & Fidell, 2007). Participants included in the models presented valid responses to all selected variables. Moderating effects were visualized by determining two thresholds (two standard deviations above and below the mean) to create three groups within the two interacting variables and then calculating the resulting happiness value, according to the final model. Data were weighted to be representative of ILF populations in Quebec, using the full 2021 census group housing data (Statistics Canada, 2021). Analyses were performed using Stata v17 (StataCorp, 2021).

## Results

The sample comprised 979 older adults, distributed almost equally between residents of conventional dwellings (CD) and independent living facilities (ILF; 51.95 vs. 48.05%). Aged between 75 and 100 and older in ILF than CD, about two thirds were women. Respondents had similar levels of happiness, regardless of their sex or setting, when adjusted for age, living with someone, education, and physical and mental health (Table 1). All happiness subscores were also similar across sex and settings, with the only difference being in negative affect between women and men in ILF. Compared to those in ILF, respondents in CD were younger, participated in fewer activities, were discriminated against more often, lived with at least one other person, had a university education, were in a relationship, drove at least once a month, and still had a full or part-time job (Table 1). Overall, compared to women, men were younger, had fewer negative affect, were more integrated into their community, reported having access to more services, were more likely to live with another person, have a university education, be in a relationship, drive a least once a month, and have a full or part-time job. In addition, more women lived in ILF than CD. In CD, men felt more integrated in their community, reported greater access to services, were more likely to be in a relationship, have university education, and a job. In IFL, women reported greater negative affect than men, more self-directed ageism. Women in both settings reported similar happiness and satisfaction with life. Respondents in both settings had similar positive affect and quality of life based on capabilities (Table 1).

**Table 1.** Description and Comparison of Respondents.

Variable	Conventional Dwelling			Independent Living Facility			Overall		
	Total (*n* = 507)	Women (*n* = 330)	Men (*n* = 177)	Total (*n* = 469)	Women (*n* = 342)	Men (*n* = 127)	Total (*n* = 976)	Women (*n* = 672)	Men (*n* = 304)
	Continuous Characteristics								
	Mean (s.d.)	Mean (s.d.)	Mean (s.d.)	Mean (s.d.)	Mean (s.d.)	Mean (s.d.)	Mean (s.d.)	Mean (s.d.)	Mean (s.d.)
Age	80.35 (4.66)	80.64^a^ (4.78)	79.84^a^ (4.40)	84.24*** (5.39)	84.58^b^ (5.16)	83.29^b^ (5.88)	82.22 (5.38)	82.64*** (5.35)	81.29 (5.34)
Happiness^c^	69.68 (5.67)	69.51^a^ (5.77)	70.13^a^ (5.45)	68.40 (5.72)	67.67^a^ (5.81)	70.13^a^ (5.43)	69.07 (5.71)	68.61 (5.80)	70.05 (5.44)
Satisfaction with life^c^	15.75 (0.95)	15.65^a^ (0.96)	15.96^a^ (0.92)	15.56 (0.95)	15.47^a^ (0.96)	15.78^a^ (0.92)	15.66 (0.96)	15.56 (0.97)	15.86 (0.92)
Positive affect^c^	7.03 (0.46)	7.14^a^ (0.47)	6.82^a^ (0.45)	7.02 (0.46)	7.04^a^ (0.47)	6.99^a^ (0.44)	7.02 (0.46)	7.09 (0.47)	6.89 (0.44)
Negative affect^c^	2.74 (0.54)	2.82^ab^ (0.56)	2.53^b^ (0.51)	2.98 (0.56)	3.22^a^ (0.57)	2.40^b^ (0.53)	2.85 (0.55)	3.02*** (0.56)	2.49 (0.52)
Quality of life based on capabilities^c^	9.96 (0.93)	9.92^a^ (0.95)	10.05^a^ (0.89)	9.76 (0.94)	9.76^a^ (0.95)	9.75^a^ (0.89)	9.87 (0.94)	9.84 (0.95)	9.91 (0.89)
Thriving	47.64 (8.27)	47.17^a^ (8.72)	47.55^a^ (7.35)	48.04 (7.24)	48.02^a^ (7.63)	48.18^a^ (6.16)	47.85 (7.81)	47.60 (8.19)	48.39 (6.87)
Social participation	22.08 (16.45)	22.31^a^ (17.33)	21.78^a^ (14.76)	24.64*** (20.54)	23.98^a^ (20.16)	26.04^a^ (21.49)	23.28 (18.56)	23.15 (18.82)	23.57 (17.99)
Community integration	34.57 (4.83)	34.08^b^ (4.77)	35.51^a^ (4.82)	34.67 (4.91)	34.62^ab^ (5.00)	34.73^ab^ (4.70)	34.62 (4.87)	34.36* (4.89)	35.19 (4.77)
Ageism (discrimination)	14.63 (4.20)	14.60^a^ (4.09)	14.62^a^ (4.37)	13.92** (4.14)	14.02^a^ (4.20)	13.66^a^ (4.01)	14.28 (4.18)	14.31 (4.16)	14.22 (4.24)
Ageism (self-directed)	8.03 (1.48)	8.10^ab^ (1.49)	7.90^a^ (1.45)	8.18 (1.60)	8.06^a^ (1.57)	8.49^b^ (1.64)	8.10 (1.54)	8.15 (1.56)	8.08 (1.53)
Services	11.95 (2.24)	11.65^a^ (2.15)	12.52 (2.31)	11.64* (2.28)	11.62^a^ (2.21)	11.70^a^ (2.50)	11.80 (2.27)	11.64** (2.17)	12.17 (2.42)

	Categorical Characteristics								
	*n* (%)	*n* (%)	*n* (%)	*n* (%)	*n* (%)	*n* (%)	*n* (%)	*n* (%)	*n* (%)
Women	330 (65.09)	n/a	n/a	340** (72.73)	n/a	n/a	672 (68.76)	n/a	n/a
Lives with someone	271 (53.35)	152^a^ (46.20)	119^a^ (67.23)	116*** (21.18)	68^b^ (19.36)	48^b^ (26.34)	387 (38.08)	220*** (32.59)	167 (50.10)
Physical health – Excellent/very good/good (vs. Average/poor)	432 (85.21)	274^a^ (83.54)	156^a^ (88.14)	386 (80.59)	277^a^ (79.88)	109^a^ (82.18)	816 (82.91)	551 (81.67)	265 (85.64)
Mental health – Excellent/very good/good (vs. Average/poor)	490 (96.27)	318^a^ (96.36)	170^a^ (96.05)	454 (96.41)	331^a^ (96.29)	123^a^ (96.65)	942 (96.32)	649 (96.33)	293 (96.30)
Education – High school	228 (45.24)	157^a^ (48.16)	70^a^ (39.77)	201 (50.42)	151^a^ (51.33)	50^a^ (48.79)	428 (47.82)	308 (49.77)	120 (43.56)
Trade school/college	92 (18.25)	61^a^ (18.71)	31^a^ (17.61)	10 (21.79)	81^a^ (23.88)	19^a^ (16.60)	192 (20.03)	142 (21.33)	50 (17.19)
University	184 (36.51)	108^ab^ (33.13)	75^b^ (42.61)	164* (27.79)	107^a^ (24.79)	57^ab^ (34.60)	347 (32.15)	215** (28.89)	132 (39.25)
Has children	433 (85.07)	280^a^ (84.85)	151^a^ (85.31)	387 (82.13)	278^a^ (82.00)	109^a^ (82.18)	818 (83.59)	558 (83.40)	260 (84.00)
In a couple	241 (47.44)	120^a^ (36.36)	121 (68.75)	130*** (24.38)	74^b^ (21.78)	56^ab^ (31.65)	371 (36.53)	194*** (28.98)	177 (53.15)
Drives at least once a month	373 (73.28)	209^b^ (63.33)	162 (91.53)	190*** (35.43)	115^a^ (30.80)	75^ab^ (48.23)	561 (55.17)	324*** (46.86)	237 (73.38)
Financial situation – Excellent or good (vs. Average, fair or difficult)	366 (72.48)	228^a^ (69.72)	128^a^ (78.41)	331 (66.60)	240^a^ (66.22)	91^a^ (68.61)	697 (69.94)	468 (67.95)	229 (74.29)
Retired	483 (95.27)	320^a^ (97.56)	161 (90.96)	461*** (99.31)	339^a^ (99.32)	122^a^ (99.27)	942 (97.20)	659** (98.46)	283 (94.44)
Urban (vs. Rural)	388 (79.67)	261^ab^ (83.39)	126^a^ (73.26)	413** (87.27)	300^b^ (86.90)	113^b^ (88.03)	800 (83.20)	561 (85.10)	239 (79.15)

S.D.: standard deviation.

*p*-values associated with *t*- and chi^2^ tests for independent samples, for continuous and categorical variables, respectively, indicating a difference between the Women and Men Overall columns, and the Total columns for conventional dwellings and ILF (***p < .001, **p < .01, *p < .05).

^a,b^Estimates for women and men in the same row that share a superscript letter (a or b) are not significantly different (p > 0.05, Bonferroni corrected, with *t*- and chi^2^ tests for independent samples).

^c^Mean is adjusted (age, lives with someone, education, physical and mental health).

**Figure 2.**
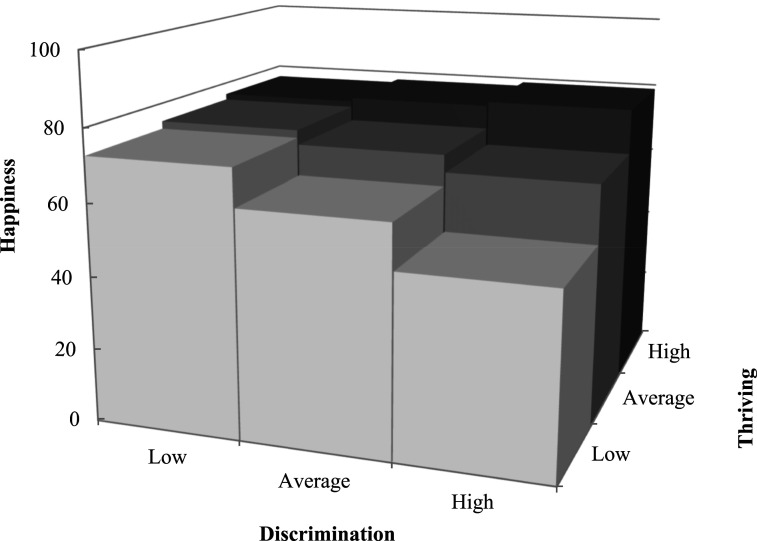
Moderating effect of discrimination on the association between thriving and happiness for older women living in conventional dwellings.

**Figure 3.**
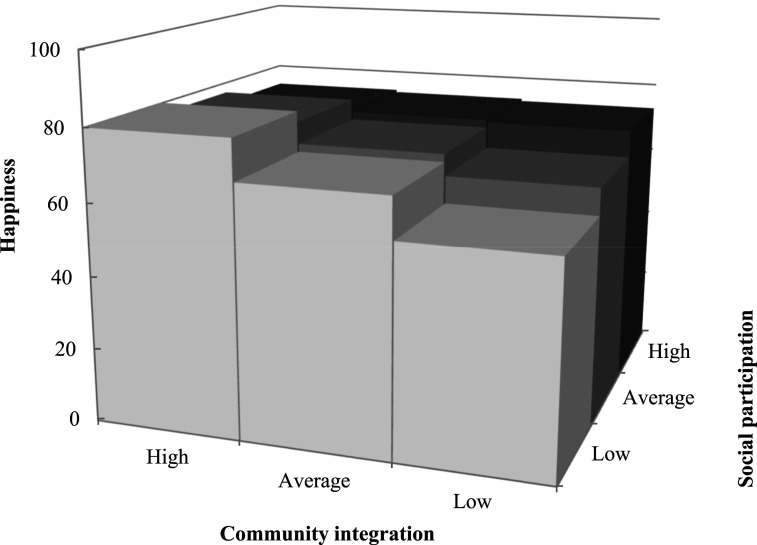
Moderating effect of community integration on the association between social participation and happiness for older women living in conventional dwellings.

For women living in CD, after controlling for personal characteristics, greater happiness was associated with more social participation (β = 0.67; *p* < .05) and community integration (β = 0.42; *p* < .01), and fewer experiences of age discrimination (β = −1.02; *p* < .01). Greater happiness was associated with more thriving in the minimal model (β = 0.47; *p* < .001), but its main effect was non-significant in the subsequent models. However, the associations of happiness with thriving and social participation were moderated by discrimination (β = 0.96; *p* < .01; Figure 2) and community integration (β = −0.69; *p* < .05; Figure 3), respectively. More specifically, when experiencing more discrimination, greater thriving was associated with more happiness, but women’s happiness decreased sharply with reduced thriving. Being highly integrated in one’s community moderated the association between less social participation and happiness. In other words, when less integrated in their community, women who participated less socially were not as happy as those with frequent participation, but this difference disappeared with greater integration. The superior happiness of women living in ILF was associated with more thriving (β = 0.58; *p* < .001). The final models explained more than half of the variance in happiness of women living in CD and ILF, according to the coefficients of determination (Table 2).

**Table 2.** Multivariate Models Estimating the Happiness of Older Women Living in Conventional Dwelling or Independent Living Facility.

	Conventional Dwelling				Independent Living Facility			
	Minimal	Inter-mediate	Adjusted	Final	Minimal	Inter-mediate	Adjusted	Final
	Beta (s.e.)	Beta (s.e.)	Beta (s.e.)	Beta (s.e.)	Beta (s.e.)	Beta (s.e.)	Beta (s.e.)	Beta (s.e.)
Thriving	0.47*** (0.08)	−0.15 (0.27)	−0.18 (0.23)	−0.18 (0.23)	0.70*** (0.12)	1.70*** (0.96)	1.28*** (0.72)	0.58*** (0.11)
Social participation	0.20** (0.38)	0.90** (2.35)	0.67* (2.28)	0.67* (2.28)	0.05 (0.43)			
Ageism (discrimination)		−1.15*** (1.04)	−1.02** (1.01)	−1.02** (1.01)				
Thriving * discrimination		1.07** (0.01)	0.96** (0.02)	0.96** (0.02)				
Ageism (self-directed)						−0.13* (0.49)	−0.10 (0.47)	
Community integration		0.51*** (0.36)	0.42** (0.35)	0.42** (0.35)		1.21** (1.26)	0.72 (1.02)	
Thriving * community integration						−2.06* (0.03)	−1.34* (0.02)	
Social participation * community integration		−0.94** (0.07)	−0.69* (0.06)	−0.69* (0.06)				
Age			0.10* (0.11)	0.10* (0.11)			0.08 (0.14)	0.08 (0.14)
Lives with someone			−0.01 (1.09)	−0.01 (1.09)			0.02 (1.98)	0.03 (2.01)
Physical health (ref = Excellent/very good/good)								
Average/poor			−0.22*** (1.92)	−0.22*** (1.92)			−0.02 (1.77)	−0.02 (1.90)
Mental health (ref = Excellent/very good/good)								
Average/poor			0.02 (3.97)	0.02 (3.97)			−0.12*** (2.17)	−0.12*** (2.27)
Income (ref = Excellent or good)								
Average, fair or difficult			−0.04 (2.16)	−0.04 (2.16)			−0.10* (2.58)	−0.09 (2.64)
Services			0.08 (0.33)	0.08 (0.33)			0.21*** (0.37)	0.24*** (0.37)
Observations	296	296	296	296	310	310	310	310
Coefficient of determination	0.292	0.447	0.514	0.514	0.518	0.547	0.615	0.600

Robust standard errors (s.e.) in parentheses. ***p < .001, **p < .01, *p < .05. Minimal model includes thriving and social participation main effects; Intermediate model adds ageism (self-directed and discrimination) and their interaction terms; Adjusted model adjusts for personal characteristics as control variables; Final model keeps studied variables significantly associated with happiness.

**Figure 4.**
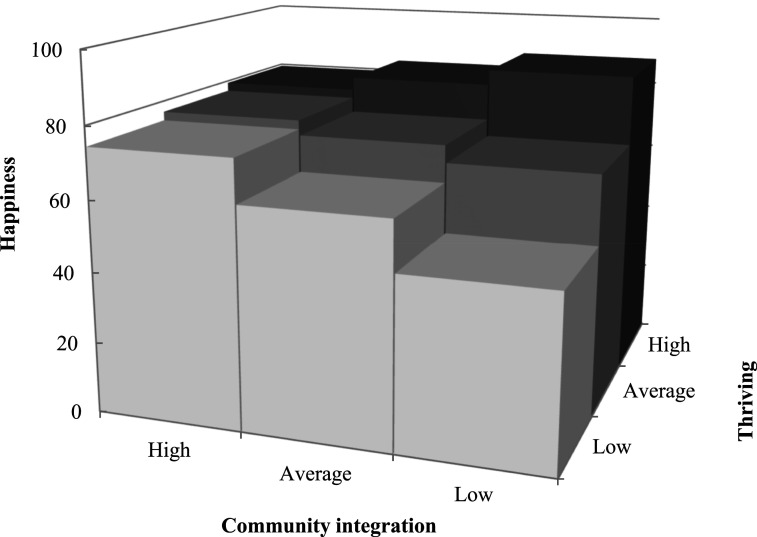
Moderating effect of community integration on the association between thriving and happiness for older men living in conventional dwellings.

For men living in CD, after controlling for personal characteristics, higher happiness scores were associated with greater thriving (β = 1.48; *p* < .01) and community integration (β = 1.18; *p* < .01), and less self-directed ageism (β = −0.21; *p* < .001; Table 3). The association between happiness and thriving was moderated by community integration (β = −1.89; *p* < .05), i.e., reduced thriving was associated with greater happiness if men were well integrated in their community. Conversely, greater thriving was associated with greater happiness, even if men were less integrated (Figure 4). For men living in ILF, greater happiness was also associated with greater thriving (β = 0.28; *p* < .001) and less self-directed ageism (β = −0.28; *p* < .01). According to the coefficients of determination, the final models explained more than half of the variance in men’s happiness in CD and almost two-third in ILF (Table 3).

**Table 3.** Multivariate Models Estimating the Happiness of Older Men Living in Conventional Dwelling or ILF.

	Conventional Dwelling				Independent Living Facility			
	Minimal	Inter-mediate	Adjusted	Final	Minimal	Inter-mediate	Adjusted	Final
	Beta (s.e.)	Beta (s.e.)	Beta (s.e.)	Beta (s.e.)	Beta (s.e.)	Beta (s.e.)	Beta (s.e.)	Beta (s.e.)
Thriving	0.64*** (0.14)	2.42*** (0.85)	1.59** (0.88)	1.48** (0.88)	0.53*** (0.32)	0.37* (0.30)	0.31** (0.24)	0.28*** (0.23)
Social participation	0.02 (0.51)				−0.07 (0.85)	−0.69* (1.97)	−0.46 (2.30)	
Ageism (discrimination)		−0.13* (0.20)	−0.09 (0.18)			−0.91 (0.81)	−0.53* (0.86)	−0.28** (0.31)
Social participation * discrimination						0.94* (0.14)	0.51 (0.16)	
Ageism (self-directed)		−0.22*** (0.52)	−0.20*** (0.54)	−0.21*** (0.52)				
Community integration		1.92*** (1.09)	1.28** (1.09)	1.18** (1.10)				
Thriving * community integration		−3.26*** (0.02)	−2.06** (0.02)	−1.89* (0.02)				
Age			0.09 (0.16)	0.09 (0.16)			−0.12 (0.22)	−0.11 (0.22)
Lives with someone			0.06 (1.67)	0.06 (1.67)			0.03 (2.79)	0.02 (2.86)
Physical health (ref = Excellent/very good/good)								
Average/poor			−0.14** (2.56)	−0.15* (2.55)			−0.28 (4.24)	−0.28 (4.17)
Mental health (ref = Excellent/very good/good)								
Average/poor			−0.06 (3.41)	−0.08 (3.32)			−0.28* (5.19)	−0.14*** (5.06)
Income (ref = Excellent or good)								
Average, fair or difficult			−0.13* (2.90)	−0.13** (3.04)			−0.06 (4.22)	−0.07 (3.86)
Services			0.07 (0.38)	0.10 (0.38)			0.25 (0.72)	0.26 (0.69)
Observations	169	169	169	169	123	123	123	123
Coefficient of determination	0.422	0.496	0.553	0.547	0.250	0.461	0.655	0.638

Robust standard errors (s.e.) in parentheses. ***p < .001, **p < .01, *p < .05. Minimal model includes thriving and social participation main effects; Intermediate model adds ageism (self-directed and discrimination) and their interaction terms; Adjusted model adjusts for personal characteristics as control variables; Final model keeps studied variables significantly associated with happiness.

## Discussion

This study aimed to compare happiness in older women and men living in CD and ILF, and examine happiness’s associations with thriving and social participation, while testing for the moderating effect of community integration and ageism (self-directed and discrimination), in accordance with ecological models (Bronfenbrenner, 1979; Fougeyrollas et al., 2019). Similar levels of happiness were found, regardless of sex or setting, which suggests that happiness is comparable across living environments in populations aged 75+, despite older respondents in ILF. This study’s findings are consistent with the results of previous studies with similar populations and residential settings, in California (Jeste et al., 2019), Croatia (Despot Lučanin et al., 2020) and Sweden (Corneliusson et al., 2020). However, in Chicago, greater satisfaction with life was reported for respondents in CD (Lynch, 2012), but using convenience sampling. Our results are also coherent with a meta-analysis of 58 studies, where happiness measured with composite indicators did not differ by sex (Pinquart & Sörensen, 2001). Previous studies found that life satisfaction was superior in older men than women, which might be partially explained by higher rates of widowerhood, higher risks of health problems and lower material resources (Joshanloo & Jovanović, 2020; Pinquart & Sörensen, 2001). In the present study, associations with happiness were controlled according to living with another person and health self-assessment, which may explain that no sex differences were observed. Thriving was associated with happiness, highlighting the importance of a better fit between the person and their environment to ensure the fulfilment of their aspirations. Superior community integration was associated with happier older adults living in CD, as well as social participation in women. Less happiness was also associated with self-directed ageism in men in CD, and with discrimination for women in CD and men in ILF.

### Thriving

Other studies confirmed the associations between happiness and thriving individuals, i.e., living in a supportive environment, with opportunities for meaningful activities and interactions with family, friends, and caregivers. According to a Swedish study (*n* = 1955 living in ILF and *n* = 1850 living in CD), quality of life based on capabilities (measured by the ICECAP-O instrument) was associated with thriving (TOPAS; *r* = 0.545; *p* < .001); there was no difference in well-being between both settings (*p* = .64; Corneliusson et al., 2020). With advanced aging, where there is a greater risk of a decline in individual abilities that makes daily activities harder to accomplish, the interaction between the person and their environment assumes greater importance in fulfilling their aspirations. In the present study, men living in CD experienced the greatest impact of thriving on happiness, including when considering their integration in the community, which suggests that a helpful and informal relationship with their support network and neighbors could offset their reduced engagement in formal and structured social participation. Higher thriving was associated with greater happiness of women and men living in ILF, and not social participation. The lack of association with participation may be attributed to increased limitations with instrumental activities of daily living, which could necessitate more support from their living environment, strengthening their relationships and care provided by the staff, with whom they can develop close bonds and friendships. According to a study with 136 Swedish older adults aged 65 to 100, living at home and receiving homecare services, greater thriving was found to be mainly associated with more social relationships, followed by greater self-determination in activities in and around the house (Lämås et al., 2020), but these results were not stratified according to sex. Thriving is fostered by the immediate social environment when it contributes to the development of social support, networks and connections, notably by providing more opportunities for social activities. A holistic adaptation of the home and surrounding environment is key to improving sociability and facilitating thriving, which supports the fulfilment of older adults’ social needs.

### Social Participation and Community Integration

In this study, higher social participation and community integration were associated with greater happiness in older adults living in CD and interacted with each other (for women) or with thriving (for men), potentially by fostering a supportive environment and opportunities for meaningful social activities (Scharlach & Lehning, 2013). Higher social participation was only associated with greater happiness in women, coherent with one study among older Portuguese individuals (*n* = 467), where being female rather than male was more associated with social participation, which in turn contributed to superior well-being (Mónico & Margaça, 2024). Another study also found that older men from Germany (*n* = 6089) were hesitant to engage in formal and organized activities, preferring those providing a sense of usefulness, which contributed to the positive perception of their aging (Schwartz et al., 2021). According to one systemic review, the social participation of older adults’ is fostered by their social networks (Townsend et al., 2021), which may be enhanced by their community integration, leading to larger and more diverse social networks (Smith et al., 2023). Although sharing similar levels of community integration as those in CD, the present study’s respondents living in ILF engaged in more activities with others, consistent with Japanese ILF residents, more likely to engage in hobbies as well as physical and learning activities than those living in CD (Kawaguchi et al., 2024). In the present study, happiness was however not associated with social participation and community integration in respondents living in ILF. This lack of association might be explained by thriving, i.e., better fit between person and their physical and especially social environment, such as staff and other residents, that could be more important than the activities themselves. Furthermore, the communal atmosphere, regular social activities such as morning coffee, lunches and events, and assistance from peers enhanced autonomy and happiness for ILF residents (Andrianova & Raymond, 2021; Taylor & Neill, 2009).

### Ageism

As either discrimination or self-directed stereotypes, ageism was associated with less happiness in women in CD and in men in both settings. According to previous studies, discrimination was associated with reduced satisfaction with life in both women and men among 15,759 Canadians aged 45 and over (but to a lesser extent in those aged 65 and over; Browning et al., 2020) and, longitudinally, reduced psychological well-being, diminished positive affect, and increased limitations with instrumental activities of daily living in 3034 aging Americans (Stokes & Moorman, 2020). A study with 2119 Australians aged 60 and over (Lyons et al., 2018) found that age discrimination was associated with reduced happiness, without interactions with sex. In the present study, when faced with age discrimination, thriving older women were more likely to be happier than those not thriving, which suggests that a better fit between individuals and their environment could increase their sense of self-efficacy, contributing to greater self-esteem when thriving (Orth & Robins, 2022) as well as better coping strategies to counter ageism. Self-directed ageism was also associated with inferior happiness for men in CD, which is consistent with finding from older men from Israel who were more likely to express negative attitudes towards aging adults (e.g., “Many old people just live in the past”) and have avoidant attitudes (Bodner et al., 2012), a strategy to distance themselves from physical decline and unwanted thoughts of death (Martens et al., 2005). Furthermore, age discrimination can happen between residents, and especially target those with physical and cognitive decline, as activities can require adaptation, which can become a limitation or diminish others’ interest (Andrianova & Raymond, 2021).

### Implications

By showing similar happiness in ILF and CD but different associated factors, the present findings can inform ILF’s management practices, community development and policy research aimed at older adults’ well-being. In line with the Framework for an Integrated Proximity Approach for Older Adults (Bérubé, 2024) adopted by the Government of Quebec, these results support having a continuum of services (e.g., healthcare, leisure, groceries, etc.) in the community. Fostering friendly and accessible communities that provides informal social structures, such as seniors centers (Aday et al., 2019), men’s sheds (Milligan et al., 2015) and community gardens (Kingsley et al., 2019), may facilitate reciprocal social exchanges (Scharlach & Lehning, 2013), which may be more even more effective in older men. Since happiness is associated with thriving older adults who participate socially and are integrated in their community, it is essential for ILF to create caring environments with social structures. Such social structures can be developed by hiring animators who motivate participation (Andrianova & Raymond, 2021), and by designs addressing psychosocial and physiological needs. For example, a configuration with social spaces (e.g., shared living rooms) closer to the apartments, which facilitates the informal socialization of residents with disabilities, as well as using these spaces during off-hours for informal social interactions (e.g., coffee or games; (Andrianova & Raymond, 2021; Campbell, 2015). Since ageist stereotypes (e.g., physical decline, being socially isolated, and feeling sad or depressed) are often perceived as part of aging and can hinder mobilization of support, it is essential to disprove myths and increase knowledge about aging and older adults (Palmore, 2001), using public campaigns, or training of health and social service professionals (Bétrisey et al., 2024) as well as homecare providers and ILF staff (Ory et al., 2003). Lastly, considering negative perceptions of older adults about ILF, especially following the pandemic (Bail, 2021), more residential alternatives are required (Rowles, 2018) to allow them to live active happy lives in their communities, including continuing care retirement communities, cohousing, accessory dwelling units and senior villages.

### Strengths and Limitations

To our knowledge, this is the first study to comprehensively measure happiness and compare older women and men in ILF and CD, in addition to examining happiness’s associated factors. This measure encompassed happiness’ cognitive and affective dimensions. The large sample size of adults aged 75 and over also provided the statistical power necessary to observe relatively small differences and effects between sexes and settings. Its limitations include the cross-sectional nature of the survey, restricting the assessment of happiness evolving with life course events. A social desirability bias was also possible, especially considering self-reported measures and with respondents in ILF, who might have been concerned about potential reprisal. However, participants were surveyed by an independent polling firm, were reassured about confidentiality, the absence of right or wrong answers, and the importance of reporting their reality as much as possible. Additionally, to increase trust about confidentiality, no identifiable information was collected, therefore it was not possible to identify the number of distinct ILF the respondents lived in. Volunteering with a religious group was considered in the questionnaire, but not church attendance, which may have lowered the measure of social participation in churchgoers. Other factors such as ethnicity and ILF services were not examined but could have contributed to a better understanding of happiness. As the survey of CD respondents was conducted using landline phones only, some older adults could not be reached. Lastly, sample size prevented analyses according to different residential settings, such as housing cooperatives, cohousing and retirement communities.

## Conclusion

According to the present study, women and men aged 75 and older living in conventional dwellings (CD) and independent living facilities (ILF) have similar levels of happiness, thriving, social participation, community integration, and ageism but greater negative affect in women in ILF. Higher thriving was identified as an important characteristic associated with greater happiness in older adults regardless of sex or residential setting. In addition, thriving mitigated the adverse effects of age discrimination for women in CD, and helped maintain greater happiness, even when men in CD reported inferior community integration. For women living in CD, superior community integration was associated with greater happiness, even with inferior social participation. The findings also raise awareness about the detrimental effects of age discrimination and self-directed ageist stereotypes, in both residential settings. Strategies such as reducing ageism are needed to ensure the happiness of older women and men. The present study supports a continuum of services in close proximity to older adults living environments, including community resources, healthcare, and stores, to increase opportunities for social participation and enhance community integration. Future studies should confirm the associations between happiness and residential settings, including longitudinal studies, accounting for life course events and personal characteristics such as length of stay and the scope of activities offered in ILF. Qualitative studies are also needed to gain a deeper understanding of how thriving and social participation contribute to happiness, and how these factors interact with community integration and ageism, across different residential settings.

## Data Availability

The participants in this study did not provide written consent for their data to be shared publicly; hence, due to the sensitive nature of the research, supporting data are not available.
